# The Influence of Metal-Doped Graphitic Carbon Nitride on Photocatalytic Conversion of Acetic Acid to Carbon Dioxide

**DOI:** 10.3389/fchem.2022.825786

**Published:** 2022-03-23

**Authors:** Pichnaree Sakuna, Pradudnet Ketwong, Bunsho Ohtani, Jirawat Trakulmututa, Thawanrat Kobkeatthawin, Apanee Luengnaruemitchai, Siwaporn Meejoo Smith

**Affiliations:** ^1^ Center of Sustainable Energy and Green Materials and Department of Chemistry, Faculty of Science, Mahidol University, Nakhon Pathom, Thailand; ^2^ Institute for Catalysis, Hokkaido University, Sapporo, Japan; ^3^ The Petroleum and Petrochemical College, Chulalongkorn University, Bangkok, Thailand

**Keywords:** carbon nitride, metal doping, photocatalysis, energy-resolved distribution of electron traps, electron spin resonance

## Abstract

Metal-doped graphitic carbon nitride (MCN) materials have shown great promise as effective photocatalysts for the conversion of acetic acid to carbon dioxide under UV–visible irradiation and are superior to pristine carbon nitride (g-C_3_N_4_ **,** CN). In this study, the effects of metal dopants on the physicochemical properties of metal-doped CN samples (Fe-, Cu-, Zn-, FeCu-, FeZn-, and CuZn-doped CN) and their catalytic activity in the photooxidation of acetic acid were investigated and discussed for their correlation, especially on their surface and bulk structures. The materials in the order of highest to lowest photocatalytic activity are FeZn_CN, FeCu_CN, Fe_CN, and Cu_CN (rates of CO_2_ evolution higher than for CN), followed by Zn_CN, CuZn_CN, and CN (rates of CO_2_ evolution lower than CN). Although Fe doping resulted in the extension of the light absorption range, incorporation of metals did not significantly alter the crystalline phase, morphology, and specific surface area of the CN materials. However, the extension of light absorption into the visible region on Fe doping did not provide a suitable explanation for the increase in photocatalytic efficiency. To further understand this issue, the materials were analyzed using two complementary techniques, reversed double-beam photoacoustic spectroscopy (RDB-PAS) and electron spin resonance spectroscopy (ESR). The FeZn_CN, with the highest electron trap density between 2.95 and 3.00 eV, afforded the highest rate of CO_2_ evolution from acetic acid photodecomposition. All Fe-incorporated CN materials and Cu-CN reported herein can be categorized as high activity catalysts according to the rates of CO_2_ evolution obtained, higher than 0.15 μmol/min^−1^, or >1.5 times higher than that of pristine CN. Results from this research are suggestive of a correlation between the rate of CO_2_ evolution *via* photocatalytic oxidation of acetic acid with the threshold number of free unpaired electrons in CN-based materials and high electron trap density (between 2.95 and 3.00 eV).

## Introduction

Photocatalysis has attracted great interest as an energy-efficient, low-cost, and relatively safe method for chemical conversions related to pollution abatement, and in the production of platform chemicals and other valuable substances (Hagfeldt & Graetzel, 1995; Hoffmann et al., 1995; Fujishima et al., 2000; Fujishima et al., 2008). Exploiting the advantages of photocatalysis requires the development of semiconducting materials having desirable characteristics such as high surface area, low electron-hole pair recombination rate, suitable band-gap energy based on light source, and the appropriate type and amount of bulk and surface defects. Several reports have focused on explaining the correlation between energy band gap and photocatalytic performance (Srikhaow & Smith, 2013; Dante et al., 2019), while other studies have probed the defect characteristics of semiconductor materials (Anantachaisilp et al., 2014; 2015). Recently, graphitic carbon nitride (g-C_3_N_4_, CN) has gained attention in the photocatalysis research field, with potential applications in the decomposition of organic pollutants, in water splitting, and for carbon dioxide reduction (Dinesh & Chakma, 2019; Li et al., 2019; Volokh et al., 2019; Wang et al., 2020; H-g.; Zhang et al., 2018). It is easy to synthesize, exhibits high thermal and chemical stability, and exhibits a moderate band gap (2.7 eV) and an absorption edge at 450 nm. Despite this, some enhanced visible light activity CN materials show a high recombination rate of photogenerated electron-hole pairs, which results in low photocatalytic performance (Dong et al., 2013). From literature, the dopants may contribute toward the suppression of recombination of charge carriers and enhancing the photocatalytic activity of carbon nitride (Barrio et al., 2021). Metal doping is one strategy for suppressing the recombination of photogenerated electron holes in semiconductors. This method is considered an effective way to extend the light absorption range, modify the electronic structure, and enhance surface properties, all of which can improve the photocatalytic activity of g-C_3_N_4_. Doping of copper into mesoporous C_3_N_4_ (mpg-C_3_N_4_) doubled the photocatalytic activity of the material relative to pure mpg-C_3_N_4_ for the degradation of methyl orange. This was due to a higher separation rate and greater mobility of the photogenerated carriers (Le et al., 2016). In addition, Fe-doping into g-C_3_N_4_ resulted in enhanced light absorption and photocatalytic activity, and the resulting material could be reused five times without any change in activity (Tonda et al., 2014). Bi-metallic doping can result in even higher photocatalytic activities, with Fe and P co-doped g-C_3_N_4_ materials being active photocatalysts for Rhodamine B photodegradation and hydrogen production (Hu et al., 2014). Higher activities for dye degradation were observed for co-doped Fe and P g-C_3_N_4_ relative to singly doped (with Fe or P) CN or undoped CN, probably due to the narrower band gap, larger specific surface area, and lower degree of polymeric condensation. Optoelectronic properties often are linked with the activity of CN photocatalysts, with less discussion on contributions from the surface and the bulk structure.

Reversed double-beam photoacoustic spectroscopy (RDB-PAS) is a newly utilized technique for investigating the surface properties of metal oxides and O/S doped g-C_3_N_4_ (Chuaicham, et al., 2020). Fingerprint energy-resolved distribution of electron traps (ERDTs) combined with conduction band bottom position (CBB) patterns have highlighted differences in surface structure among heteroatom-doped g-C_3_N_4_ samples, which could not be observed by other standard methods such as powder X-ray diffraction (PXRD) and infrared spectroscopy. Unpaired electrons in the g-C_3_N_4_ structure, as detected by electron spin resonance (ESR), were reported to be related to the materials’ photocatalytic activity (Li et al., 2018; Xia et al., 2019). These findings not only provide important surface structural information but also describe the relationship between structural features and photocatalytic activity (Chuaicham, et al., 2020; Nitta et al., 2018; Nitta et al., 2019). In this work, the bulk and surface structural properties of single- and bi-metal-doped g-C_3_N_4_ powders, synthesized by a facile method, were elucidated using complementary techniques with the aim to explore relationships between these and the photoactivity of these materials for the degradation of acetic acid. Acetic acid emissions contribute negatively to global warming (Budsberg et al., 2020), and this chemical is a typical degradation product from wastewater remediation (Badawi et al., 1992). While this acid is of low toxicity, it is quite stable, being oxidation-resistant under ambient conditions (Li et al., 1991). This work chose acetic acid as a model compound of a persistent organic compound in the photocatalytic conversion of acetic acid to carbon dioxide in order to represent a highly effective photocatalyst in oxidation processes.

## Materials and Methods

### Materials and Reagents

All chemicals used in this work were of analytical grade. Crystalline urea (Kemaus, Australia) was used for the preparation of g-C_3_N_4_ (CN). Copper (II) acetate monohydrate [Cu(CH_3_COO)_2_‧H_2_O] (Chem-supply, Gillman, Australia), zinc acetate dihydrate [Zn(CH_3_COO)_2_‧2H_2_O] (Univar, IL, United States), and anhydrous iron (II) acetate [Fe(CH_3_COO)_2_] (Aldrich, Auckland, New Zealand) were used without any purification as metal sources. Acetic acid was purchased from Wako Pure Chemical Industries (Japan). In addition, 65% nitric acid (HNO_3_) was purchased from RCI Labscan (Thailand) with 70% perchloric acid (HClO_4_) being obtained from Qrec, New Zealand.

### Preparation of CN and Metal-Doped CN

All samples were prepared as in a previous report (Dante et al., 2021), which utilized metal-doped carbon nitride as a sensing material for glucose. In the first step, pristine CN was prepared by the pyrolysis of urea. In a typical preparation, 25 g of urea was placed in an alumina crucible with a cover and was calcined at 873 K in an air atmosphere for 4 h (controlled heating/cooling rates of 25 K min^−1^). After cooling, the product (CN) was ground to obtain a fine powder. Subsequently, metal-doped CN was synthesized by ultrasonic impregnation, applying ultrasonic irradiation to a suspension of CN (416 mg) in aqueous metal acetate solution (33.2 mg of the appropriate metal acetate in 10 ml of deionized water). Each suspension was sonicated for 2 h and then filtered and washed with water several times. The resulting samples were dried in an oven at 338 K for 24 h to remove water, ground to a powder, and then stored in a desiccator. The obtained metal-doped CN samples were denoted as Fe_CN, Cu_CN, and Zn_CN, depending on metal dopant. The preparation of co-metal-doped CN (FeCu_CN, FeZn_CN, and CuZn_CN) followed the same procedure, dispersing CN into the aqueous solution containing 16.6 mg of each metal acetate.

### Material Characterization

X-ray diffraction patterns were recorded on an X-ray diffractometer (XRD, SmartLab, Rigaku) with Cu K*α* radiation over a 2*θ* scan range between 10° and 80°. Solid stage electron spin resonance (ESR) (Bruker ELEXSYS, ER083CS) measurements were obtained at room temperature. For this, a 20-mg portion of each sample was transferred to an ESR tube to run in X-band, at a microwave power of 20 mW, a microwave frequency of 9.850 GHz, a modulation amplitude of 1 G, and a modulation frequency of 100 kHz. ICP-MS measurements involved adding 5 mg of metal dopant CN sample in 10 ml of 1:1 v/v mixed acid solution (HNO_**3**_/HClO_**4**_) and standing overnight to ensure complete dissolution prior to analysis (ICP-MS, PerkinElmer NexION^®^ 2000 instrument). Additionally, the elemental composition of materials was analyzed using an XPS spectrophotometer (Kratos, Axis Ultra DLD) and EDX equipped with a scanning electron microscope (FE-SEM, Hitachi, SU-8010). FE-SEM imaging utilized the following instrument settings: 5.0-kV electron-acceleration voltage, 10.0-μA current, and 3.0-nm working distance. Each sample was coated for 10 s with gold using an ion sputter coater (JFC-1600, JEOL). Specific surface area (SSA) and pore size distributions of metal-doped CN samples were determined based on nitrogen (N_2_) adsorption-desorption isotherms at 77 K (Autosorb-6, Quantachrome Instrument). Prior to analysis, surface moisture was evaporated by pre-heat treatment (353 K, 2 h). The surface morphologies of the samples were also conducted on a field-emission scanning electron microscope (FE-SEM, JSM-7400F, JEOL) in the secondary-electron image mode.

ERDT/CBB patterns were measured by RDB-PAS analysis following a previous report (Chuaicham, et al., 2020). Samples were placed into a PAS cell, and prior to measurement, N_2_ saturated with methanol vapor was flowed through the cell for 30 min. The sample cell was then moved to an acrylic box under N_2_ flow and irradiated using a light beam from a xenon lamp (Bunkokeiki, Tokyo, Japan, BK1) equipped with a grating monochromator from 650 to 350 nm. The obtained PAS signal, generated by simultaneously irradiated 35-Hz modulated 625-nm LED light, was detected using a digital lock-in amplifier. Photoacoustic spectra were then recorded with reference to the photoacoustic spectrum of graphite.

The optical properties of CN-based materials were investigated using a UV–vis diffuse reflectance spectrometer (UV–vis DRS, V670, Jasco), with barium sulfate as a reference. Photoluminescence analysis (PL) utilized a photoluminescence spectrofluorometer (Horiba: FluoroMax4), with an excitation wavelength of 320 nm and an excitation slit/emission slit ratio of 2:1. Photocatalytic activity measurements.

A 30-mg portion of photocatalyst was suspended in 5 ml of 5% v/v acetic acid solution in a Pyrex tube sealed with a rubber septum. Photoirradiation was performed using a mercury arc lamp (Eiko-sha 400) at a wavelength > 290 nm. The estimated UV light (365 nm) flux was *ca.* 1 μmol s^−1^ for a 5-ml suspension. Prior to UV light irradiation, the suspension was stirred in the dark for 60 min to reach an adsorption–desorption equilibrium and then sonicated for 30 s to prevent agglomeration. While maintaining an anaerobic atmosphere, the reaction mixture was then irradiated while regulating the temperature of the vessel at 298 K with a water circulation system. The amount of generated CO_2_ was determined at 30-min time intervals using gas chromatography (GC, GC-8A, Shimadzu).

## Results and Discussion

### Chemical Composition

**FIGURE 1:**
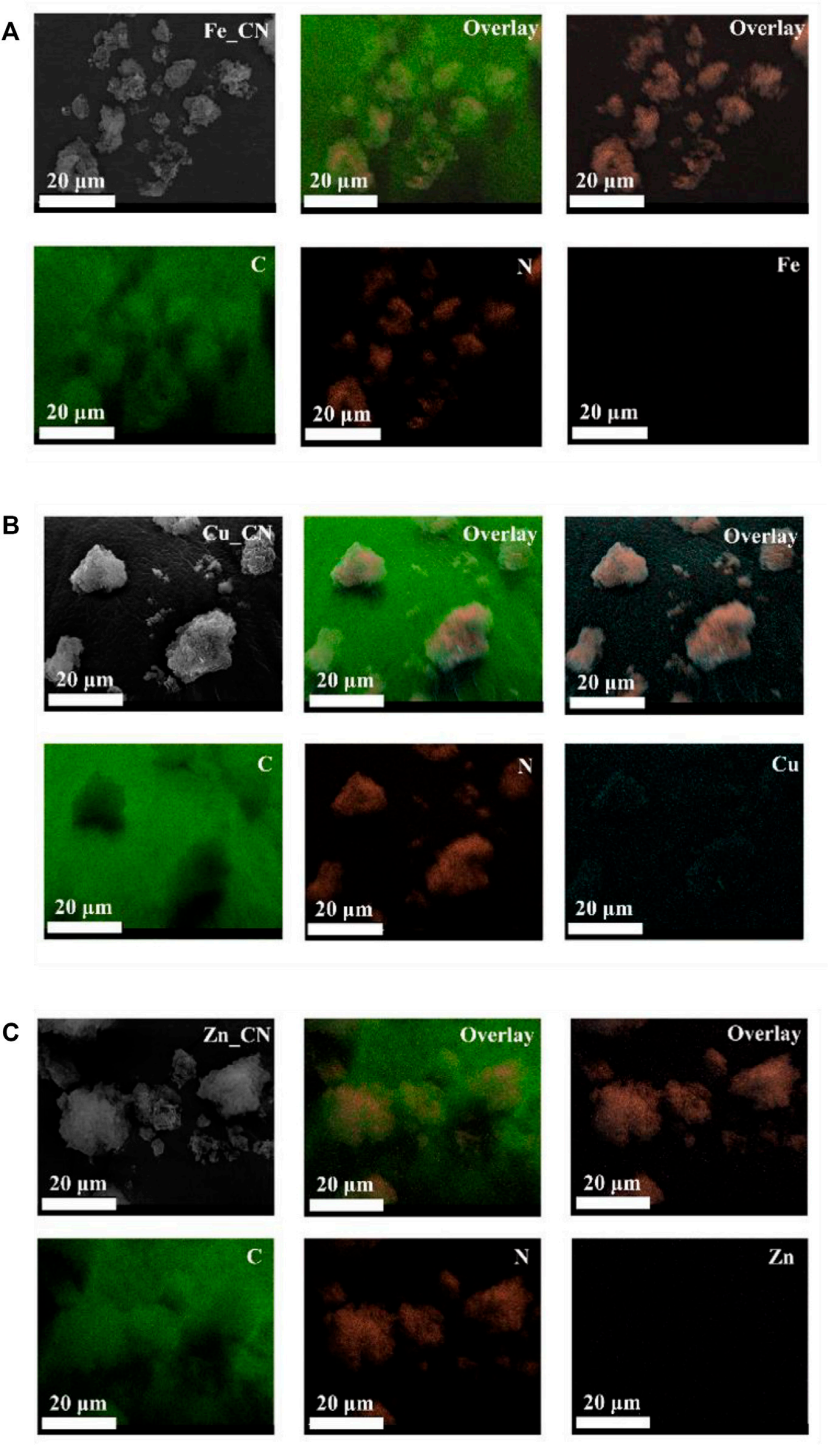
EDX mapping for single metallic doping systems **(A)** Fe_CN, **(B)** Cu_CN, and **(C)** Zn_CN.

**FIGURE 2:**
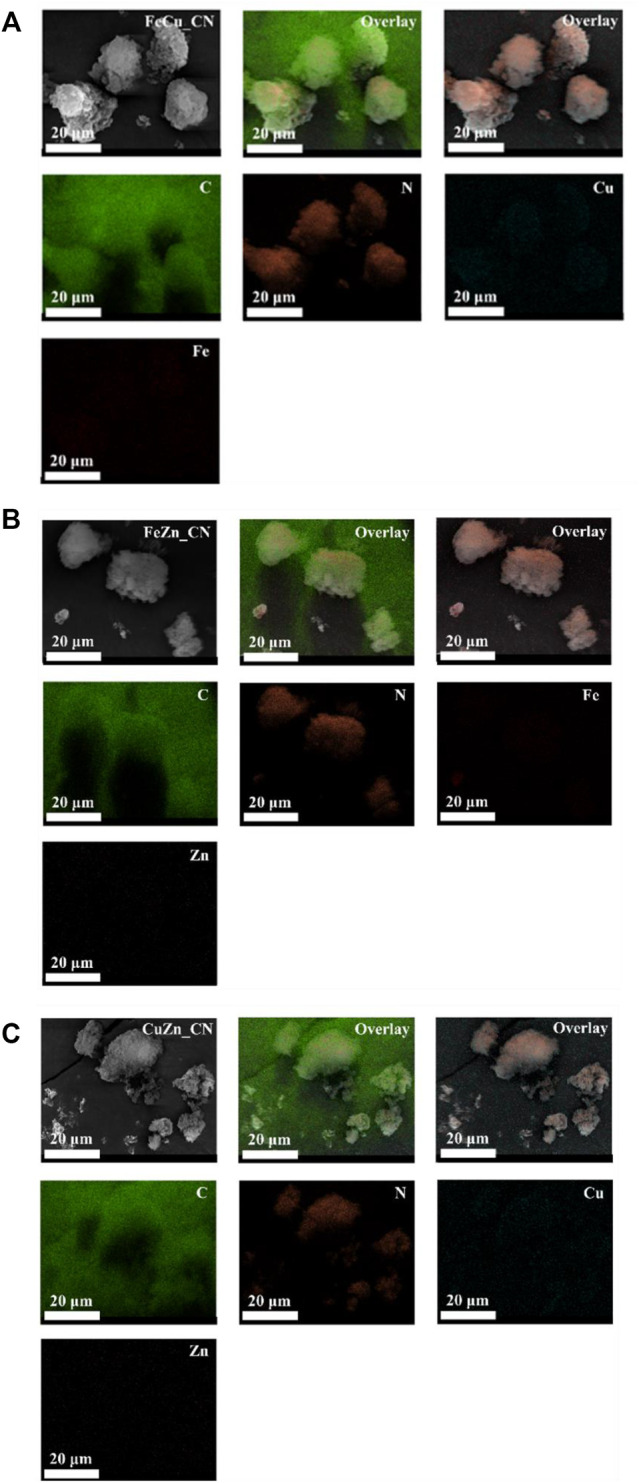
EDX mapping for bi-metallic doping systems **(A)** FeCu_CN, **(B)** FeZn_CN, and **(C)** CuZn_CN.

The metal compositions of CN-based samples, obtained using three techniques (EDX, XPS, and ICP-OES), are reported in Table 1. SEM-EDX mapping showing the localization of elements in CN-based materials is highlighted in Figures 1, 2. The images indicate the presence of very low levels of metal incorporation on the surface of all samples, while XPS spectra (Supplementary Figure S1) indicate that the surface concentrations of metals could be lower than XPS detection limits. Similar with previous studies (Praus et al., 2020; Lin et al., 2021) that reported the presence of hydroxyl groups on the CN sheets, oxygen was found in all samples (EDX mapping images). Oxygen-containing CN materials have shown high activity in photocatalytic hydrogen evolution (Huang et al., 2020) and photocatalytic dye degradation (Praus et al., 2020). On the other hand, the bulk concentrations of metal (s) in the samples were quantified by the ICP-OES technique and are reported in Table 1. The detectable % Fe (0.076 ± 0.021) and Zn (0.021 ± 0.016) in the pristine CN could be due to trace metals in the urea precursor. Also, trace metals in the iron (II) acetate, copper (II) acetate, and Zn (II) acetate reagents result in the added-up metal amount being detected in metal-doped CN materials, being relative to those in the pristine CN. No copper is detected in CuZn_CN probably due to the lower copper affinity in the CN structure.

**TABLE 1:** Comparative elemental composition of CN-based materials by SEM-EDX, XPS and ICP-MS analyses.

Material	Element (wt.%)								
	EDX			XPS			ICP-MS		
	Fe	Cu	Zn	Fe	Cu	Zn	Fe	Cu	Zn
CN	—	—	—	—	—	—	0.08	—	0.02
Fe_CN	0.02	—	—	—	—	—	0.57	—	0.08
Cu_CN	—	0.79	—	—	—	—	0.10	0.87	0.12
Zn_CN	—	—	0.06	—	=	0.39	0.20	—	0.36
FeCu_CN	0.09	1.23	—	—	—	—	1.02	0.28	0.28
FeZn_CN	0.06	—	0.04	—	—	—	1.17	—	0.03
CuZn_CN	—	0.84	—	—	—	—	—	—	0.06

### Bulk Structure

**FIGURE 3:**
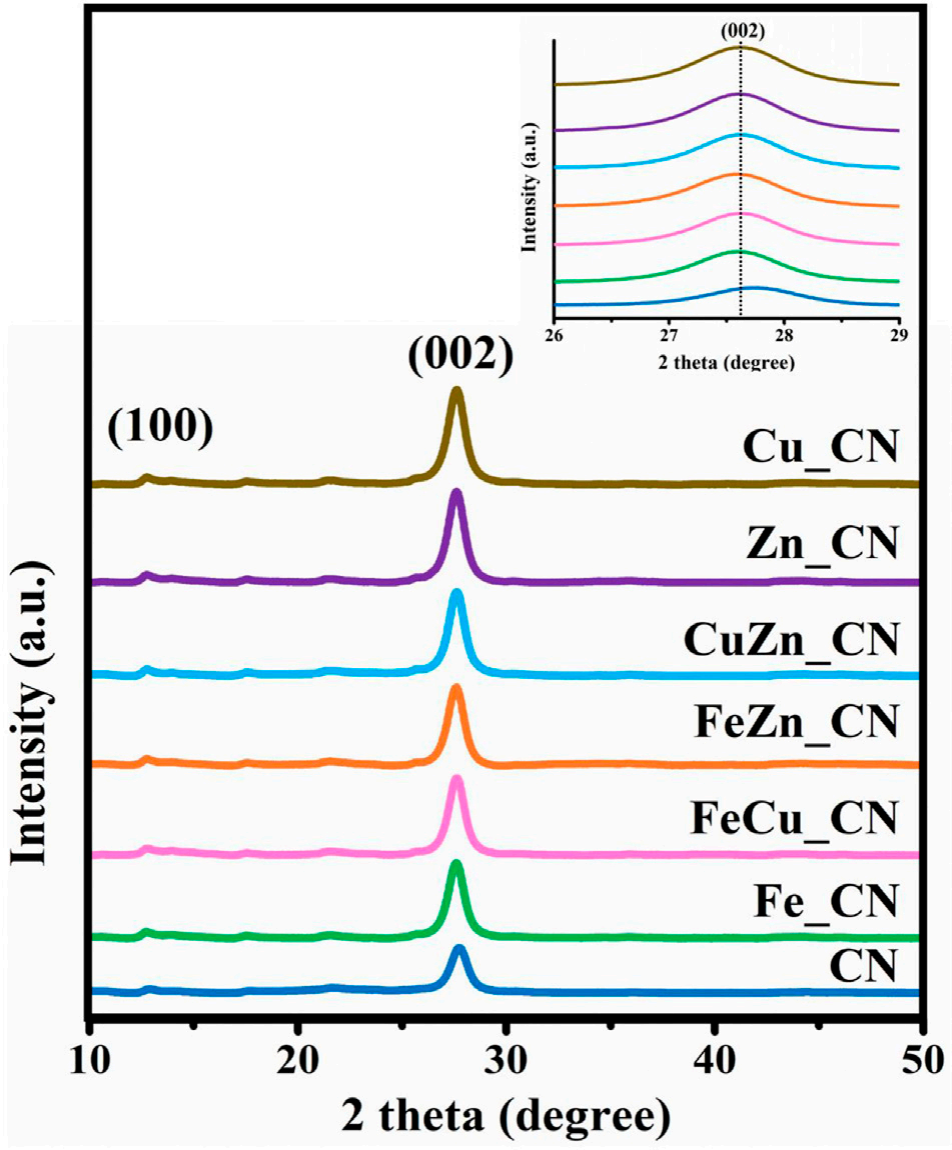
XRD patterns of undoped CN and metal-doped CN, and an enlarged view of the (002) peak.

A pristine CN sample was synthesized by urea pyrolysis at 873 K in air by modification of an established procedure (Xu et al., 2013; Maeda et al., 2018). Powder X-ray diffraction (PXRD) patterns of all CN-based samples, shown in Figure 3, exhibit a major peak at 27.7° corresponding to the (002) planes with an interlayer distance of 0.322 nm, which arises from typical interlayer stacking of hexagonal CN, and a minor peak at 13° from the (100) planes with an interplanar distance of 0.680 nm arising from in-plane structural packing motifs (Hu et al., 2014). Apart from those of pristine CN and metal-doped CN, no impurity peaks were detected even though the pyrolysis time was shorter than that used in the literature. The high intensities of the (100) and (002) peaks in the metal-doped CN samples (Fe_, FeCu_, FeZn_, CuZn_, Zn_ CN, and Cu_CN) indicated their improved crystallinity relative to undoped CN. No PXRD peaks corresponding to metals, or metal oxides, were detected, as expected due to the low metal loadings (shown in Table 1). Nevertheless, a slight shifting of the (002) peak to a lower 2-theta angle was observed for metal-doped CN samples, which suggested some modification of the graphitic stacking of CN as a consequence of metal doping, resulting in an increased interlayer distance (Zhang et al., 2018). However, varying the metal dopant does not result in any significant differences in the diffraction patterns.

**FIGURE 4:**
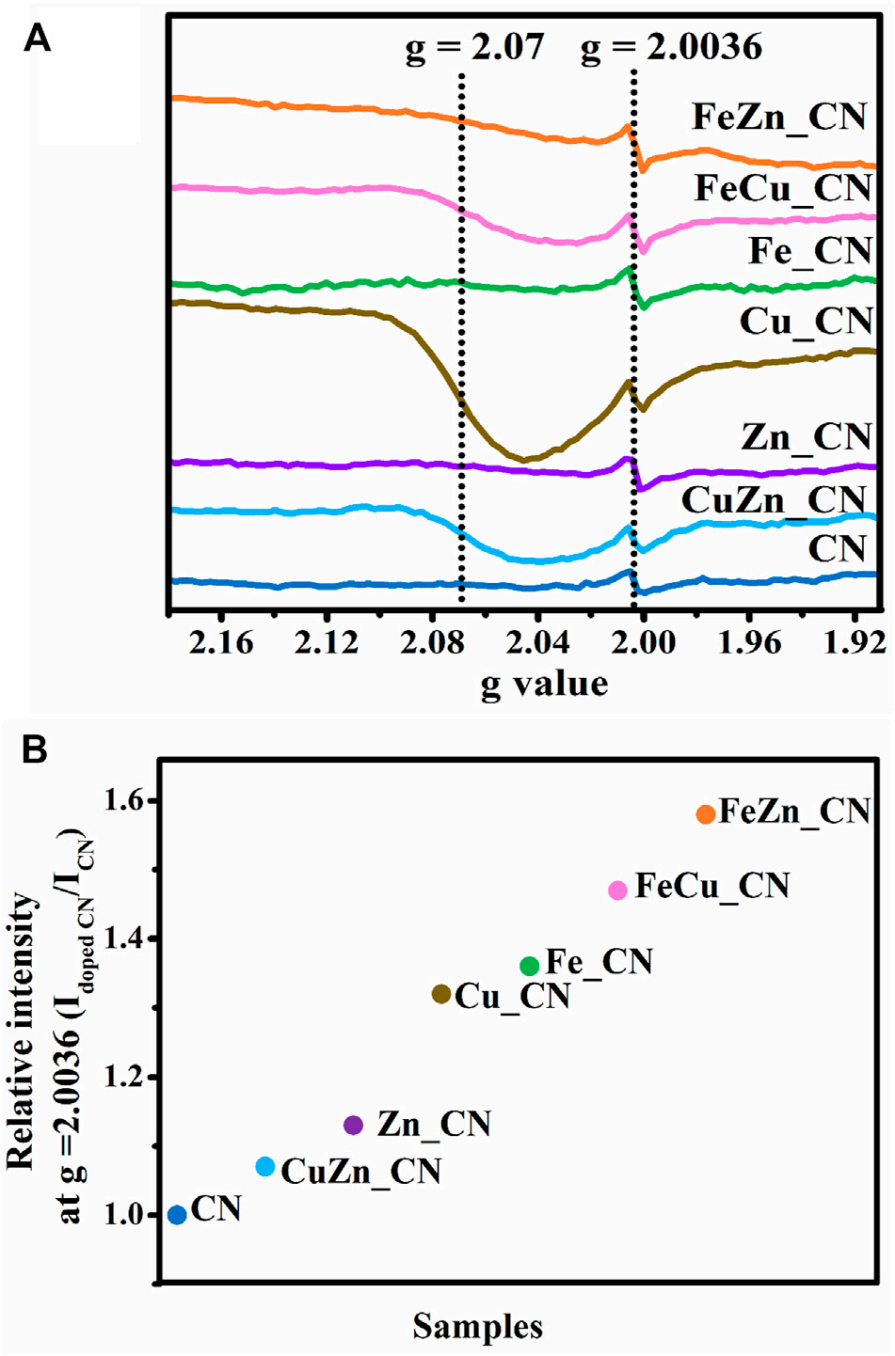
**(A)** ESR spectra of undoped CN and metal-doped CN at room temperature and **(B)** increase in relative intensity at *g* = 2.0036.

Solid-state ESR spectra, shown in Figure 4A, contain a symmetric ESR signal at *g* = 2.0036 with respect to delocalized free electrons on the aromatic ring of the CN materials (Zhang et al., 2013; Dante et al., 2021). The higher intensity of such ESR signals in metal-doped CN samples, in comparison to those seen in pristine CN, is consistent with previous work (Liu et al., 2017; Zheng et al., 2018). This indicates that the electron density or charge mobility in metal-doped CN samples is greater than in undoped CN (Chen et al., 2019). An additional ESR signal at *g* = 2.07, corresponding to Cu^2+^, can be referred to the Spin-Hamiltonian parameter along the *x* and *y* axes (*g*_xx_ = *g*_yy_ = *g*_⊥_) (Arizaga et al., 2008). The Spin-Hamiltonian parameter in the z-direction (*g*_*z*z_ = *g*_||_) around *g* = 2.3 (Arizaga et al., 2008), and the hyperfine coupling constant (A_||_), could not be detected for Cu_CN. Signals from Fe^2+^ and Zn^2+^ were also undetectable possibly due to the amount of these metal dopants being lower than the ESR detection limit. The relative signal intensity ratios for metal-doped CN/pure CN at *g* = 2.0036 are plotted in Figure 4B with the result that doping with Fe appears to afford CN-based materials containing more free unpaired electrons than is the case with other metal dopants or CN alone. The Fe-containing CN materials, FeZn_CN, FeCu_CN, Fe_CN, are ranked first, second, and third in terms of the number of free unpaired electrons in the CN-based materials.

### Surface Structure

**FIGURE 5:**
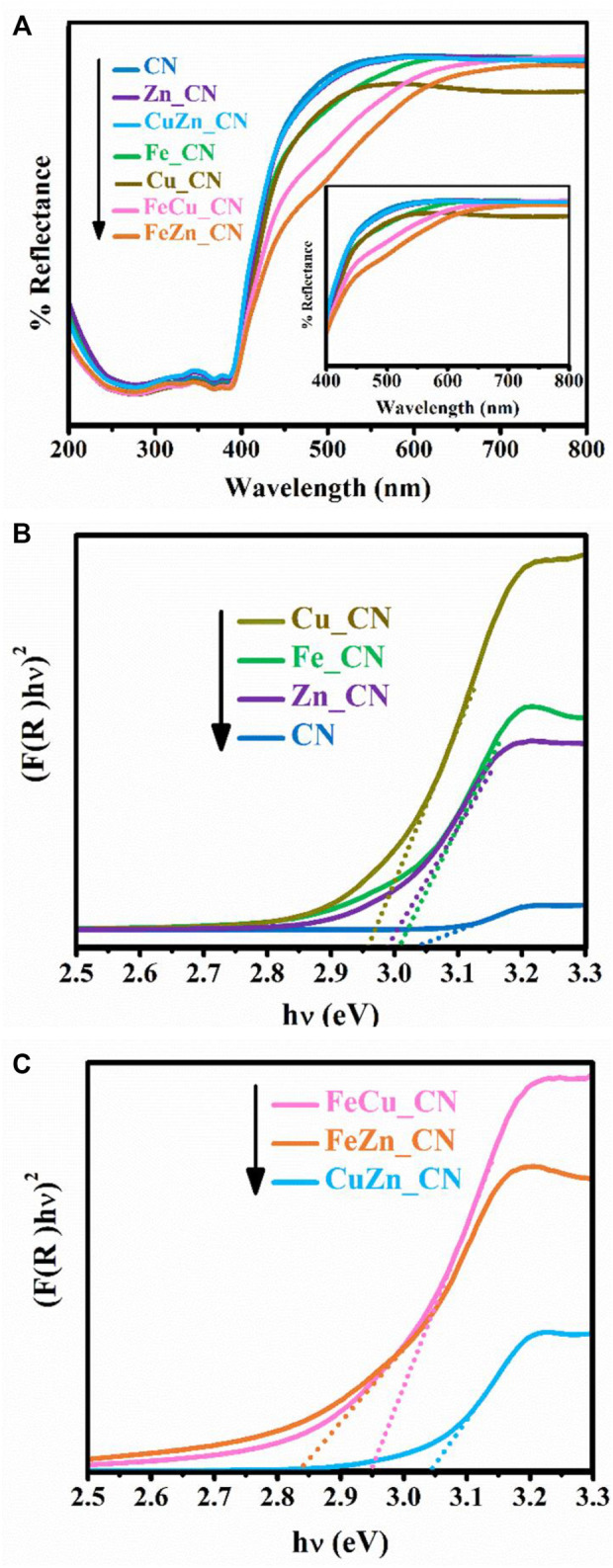
**(A)** UV–Vis diffuse reflectance spectra and estimation of band-gap energies of undoped CN, **(B)** single-metal-doped CN, and **(C)** co-metal-doped CN samples.

Since photocatalytic reactions occur on the material surface, an understanding of the surface properties is of paramount importance. UV–Vis diffused reflectance spectra of all samples are shown in Figure 5A, and these reflectance spectra can be transformed to the corresponding absorption spectra by applying the Kubelka–Munk function (Jones et al., 2019). The obtained absorption spectra, given in Supplementary Figure S2, indicate that pristine CN and metal-doped CN samples absorb light in both the UV and visible regions. Pristine CN semiconductors show an absorption range from 200 to 450 nm, which originates from charge transfer from the valence band populated by the N 2p orbital to the conduction band formed by the C 2p orbital (Wang et al., 2020). The band-gap energies of CN and CuZn_CN materials were found to be slightly higher than 3.0 eV, which is in line with the results obtained by Dante et al. (2021) and Katsumata et al. (2019) for metal-doped CN. The extended absorption spectra of metal-doped CN materials shifted into the visible range (400–50 nm), as seen in other reports (Dante et al., 2021; Liu et al., 2017; Nguyen Van et al., 2020). On the other hand, the absorption characteristics of CuZn_CN were found to be similar to pristine CN. The FeZn_CN sample showed the most optical response absorbance in the visible region, while the pristine CN showed the lowest response. The band-gap energies (E_g_) of metal-doped CN materials (Table 2), as estimated from Tauc plots (Figure 5B,C), were slightly smaller than that of CN. The CN-based materials containing Fe show enhanced light absorption and narrowing of the energy band gap (FeZn_CN; *E*_g_ = 2.84 eV), whereas those with Cu loadings exhibit a relatively high visible light response, in comparison to other metal-doped CN systems (Supplementary Figure S2). Photoluminescence (PL) results, as reported in Supplementary Figure S2, were obtained using an excitation wavelength of 320 nm. The PL spectrum of CN (Supplementary Figure S2) displays an emission peak around 457 nm (2.7 eV) caused by the transition of lone pairs to the π* conduction band (Guo et al., 2016). Results indicate that PL spectral intensities for metal(s)-doped CN spectra are significantly lower than that of pristine CN. A possible reason for this is trapping of photogenerated electrons by metal-doping sites, which leads to slower electron-hole recombination rates (Nguyen Van et al., 2020; Cui et al., 2021). Nevertheless, XPS spectra (Supplementary Figure S1) suggested the very small amount of metal incorporated on the CN surface, insufficient to be detected by the surface characterization technique. While these results agree well with those from SEM-EDX investigations (Table 1), these limitations prevent the elucidation of any interactions between metal dopants and CN sheets.

**TABLE 2:** Physical properties and photocatalytic activities of CN and metal-doped CN. In this work, two groups of materials were classified based on their high (green) and low CO_2_ evolution (black).

Material	Eg/eV	SSA/m^2^ g^−1^	Average pore size/nm	Average pore volume/cm^2^ g^−1^	d_ET_ ^a^	d_ET_ ^b^	CO_2_ evolution/µmol h^−1^
CN	3.10	91.89	3.8	0.153	9	0.0678	7.51
Fe_CN	3.00	88.45	3.8	0.153	5	0.2638	13.09
Cu_CN	2.97	84.53	4.1	0.153	3	0	7.93
Zn_CN	2.98	80.49	3.9	0.153	8	0.0678	6.70
FeCu_CN	2.94	87.58	11.3	0.153	5	0.1995	11.68
FeZn_CN	2.84	89.72	3.9	0.153	9	0.3355	13.37
CuZn_CN	3.04	97.72	3.9	0.154	4	0.0649	5.86

a The total density of electron traps (d_ET_) in the unit of μmol g^−1^.

b The d_ET_ in the range of 2.95–3.00 eV in the unit of μmol g^−1^.

**FIGURE 6:**
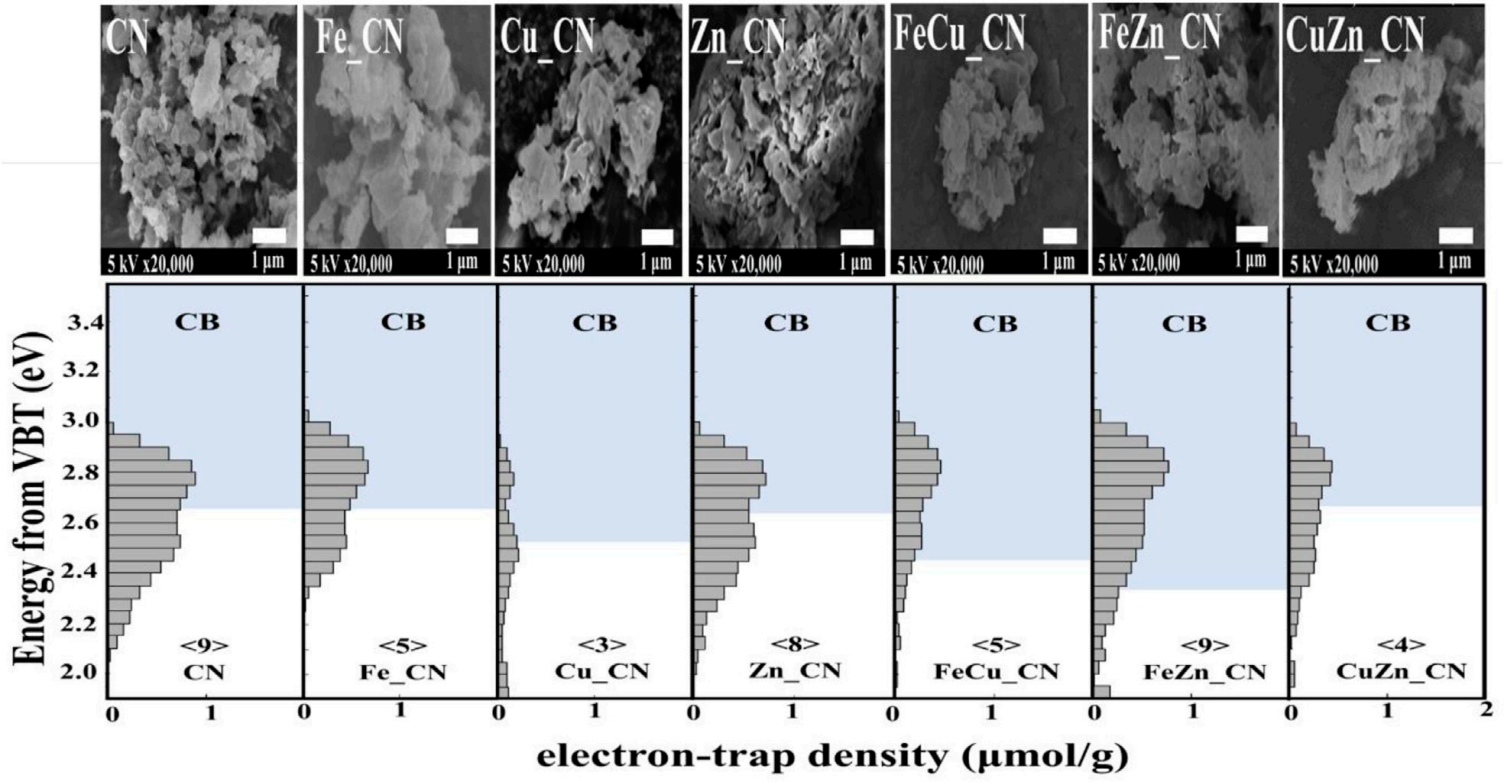
ERDT patterns with CBB positions of CN and metal-doped CN samples. The number in < > denotes the total density of ETs, with the units of μmol g^−1^. VBT, valence band top.

Nitrogen adsorption–desorption isotherms were utilized to analyze the surface of CN materials. As shown in Supplementary Figure S3 and Table 2, the specific surface area (SSA) and porosity of metal-doped CN materials vary with the dopant system. CuZN_CN has the highest SSA (97.72 m^2^ g^−1^), with all other metal-doped CN exhibiting lower SSA than pristine CN. The adsorption isotherms of pristine CN and metal-doped CN materials are given in Supplementary Figure S3, and all can be classified as type IV isotherms, which indicates the presence of mesopores. Average pore sizes and pore volumes, as calculated by the Barrett–Joyner–Halenda method, are summarized in Table 2, with FeZn_CN having an average pore size of 11.3 nm, about threefold larger than other CN materials. Figure 6 highlights the morphologies of undoped and metal-doped CN particles. All samples are composed of randomly packed thin sheet structures, constructed from the aggregation of micron-size plate-like particles, which agreed well with the observed H3 hysteresis nitrogen adsorption–desorption isotherms. The introduction of metal dopants does not appear to impact sample morphology, as indicated by the similarities in particle images. However, as stated earlier, metal doping results in slight shifting of peaks in PXRD patterns (Figure 3) along with variations in ESR signal intensities (Figure 4) and optical properties (Figures 5, 6) in CN materials, which underlines the effect of low-level metal dopants on the material structure.

Reversed double-beam photoacoustic spectroscopy (RDB-PAS) can provide information about electron accumulation in electron traps (ETs) near the conduction band bottom (CBB) on the surface of CN-based materials. Figure 6 highlights a plot of the distribution of electron traps (ERDT) versus CBB patterns, with ERDT and CBB reflecting the surface and bulk structure, respectively, of the CN-based materials. With the similar CBB, it can be concluded that the introduction of metal dopant (s) gives no effect to the bulk structure, detecting similar CBB for CN, Zn_CN, Fe_CN, and CuZn_CN materials. On the other hand, the modified bulk structures of Cu_CN, FeCu_CN, and FeZn_CN were obtained after the metal-doping process. The positions of ERDTs of Zn_CN are similar to those of pristine CN indicating neither bulk nor surface structure disruption from the introduction of Zn into the CN structure. Note that extended ERDT positions, at around 2.0 eV, were observed in the case of Cu_CN, CuZn_CN, and FeZn_CN, reflecting the modified surface structure due to metal doping. With the figures in⟨ ⟩ denoting the total density of ETs in the unit of μmol g^−1^, there is no correlation between the specific surface area (SSA) and the density of ETs found in CN-based materials, inconsistent with Nitta’s work (Nitta et al., 2019) that reported the high density of ETs for the high SSA samples. The high-density state of ETs found in FeZn_CN is the same as that of the pristine CN material. The high-density state reflecting the high number of accumulated electrons in ETs is around 2.8 eV for the pristine CN, being localized above CBB for most samples except Cu_CN. The Cu_CN material has the high-density state below CCB (0.3 eV higher energy than that of the pristine CN). Therefore, the presence of accumulated electrons in ETs between the energy band gap can suppress the electron-hole pair recombination in photocatalysts. Nevertheless, electrons may be preferably exited from the high-density state that localized below CBB.

### Photocatalytic Oxidation of Acetic Acid

The photocatalytic activity of CN-based materials toward the oxidation of acetic acid to carbon dioxide (CO_2_) was examined (Mozia et al., 2010; Nosaka et al., 1996; Ohtani et al., 2008). Carbon dioxide evolution rates for processes employing different CN materials are summarized in Table 2, and these indicate that Fe doping significantly enhances the activity of CN-based photocatalysts. Acetic acid decomposition over Fe_CN, FeCu_CN, and FeZn_CN generated CO_2_ at a rate 1.6–1.8 times higher than that of pristine CN. The highest CO_2_ evolution rate (13.37 μmol h^−1^), obtained from FeZn_CN, mirrors results obtained by Yuan et al. (2002) who found that Fe- and Zn-doped titania is far more effective as a photocatalyst for phenol degradation than single metal-doped titania alone. However, from Table 2, Cu_CN and Zn_CN give higher CO_2_ evolution rates than CuZn_CN, so it cannot always be concluded that the addition of two metal dopants gives superior performance. Although doping of Fe into CN affords a high activity CN-based photocatalyst system, there is no clear connection between the rates of CO_2_ evolution obtained from such photocatalysts and the other properties listed in Table 2.

### Property Correlations

Correlations between the activity of photocatalysts and their properties are not easy to establish with certainty. For example, a CN material having high crystallinity was reported as a promising photocatalyst due to its low resistant carrier transfer, extended light absorption range, and suppressed electron-hole pair recombination (Yang et al., 2021). However, metal-doped CN materials with relatively low crystallinity have also shown enhanced photocatalytic activity relative to pristine CN (Wang et al., 2009; Hu et al., 2014). Other properties (optical and electronic) of semiconducting materials may also significantly influence the activity of photocatalysts (Hu et al., 2014; Tonda et al., 2014; Li et al., 2016).

**FIGURE 7:**
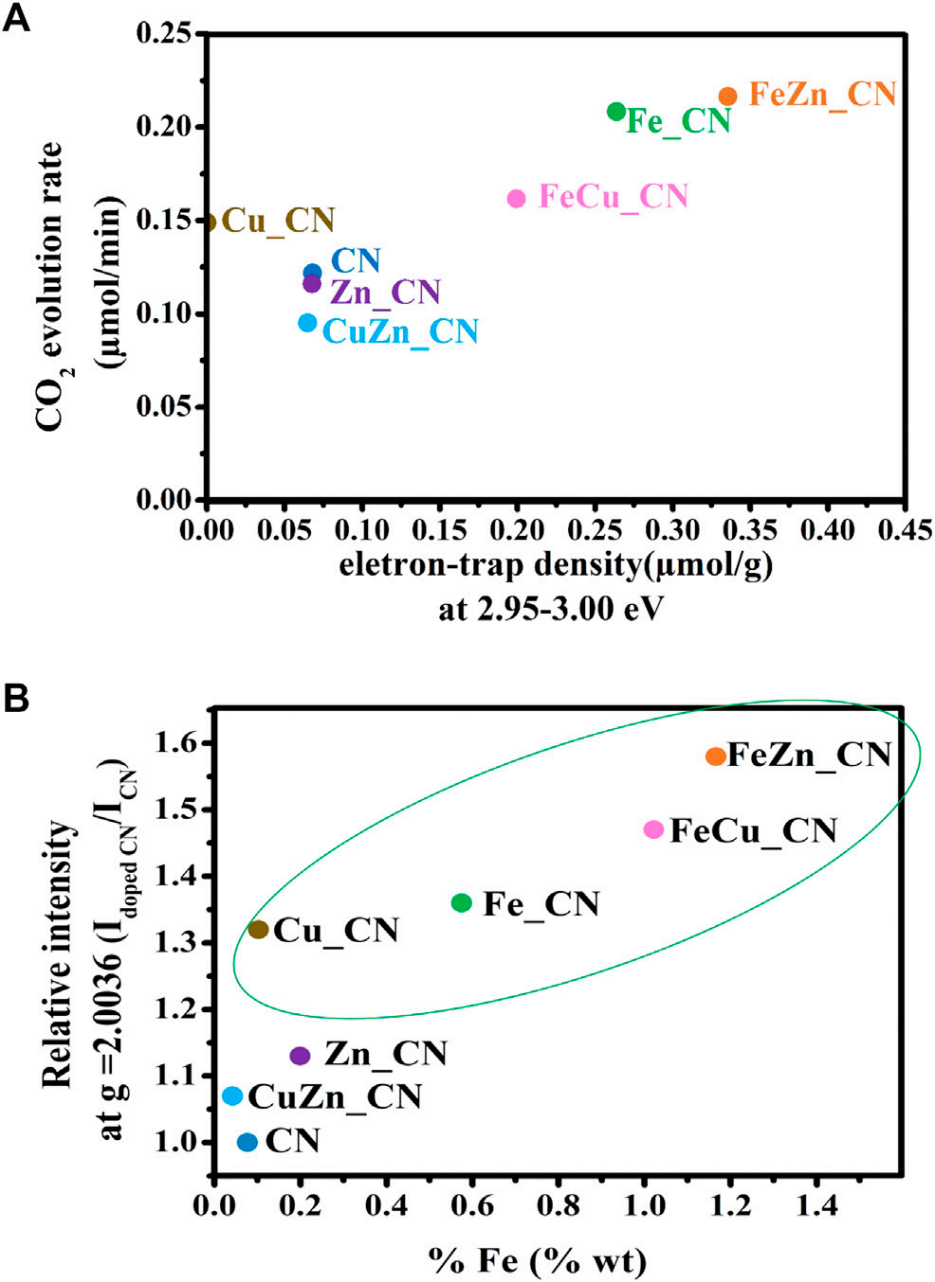
**(A)** Plot of CO_2_ evolution rate vs electron-trap density at 2.95–3.00 eV, and **(B)** the correlation between ESR signal relative intensity at *g* = 2.0036 and the bulk concentrations of Fe in carbon nitride-based materials. The materials in the green oval gave high CO_2_ evolution rates, as defined in Table 2.

Results herein indicate that the highest CO_2_ evolution rate was obtained from photocatalysis using FeZn_CN, even though Cu_CN shows the highest level of crystallinity. There is no clear correlation between all parameters related to CN materials: crystallinity, optical properties (PL, DRS, band-gap energies), specific surface area, and photocatalytic activity. Furthermore, a plot of the CO_2_ evolution rate from acetic acid oxidation under UV irradiation and electron-trap density at 2.95–3.00 eV is given in Figure 7A. From this, CN-based materials can be categorized into two groups. The first group, giving high CO_2_ evolution rates, includes Cu_CN, and Fe-containing CN materials and the rest giving low CO_2_ evolution rates are included in the second group. Figure 7B shows that the bulk concentration of Fe in samples correlates well with the relative intensity of the ESR signal at *g* = 2.0036. This correlation can thus be used to classify materials as high activity photocatalysts for conversion of acetic to carbon dioxide (as shown in the green oval). Similar correlations with respect to Cu or Zn bulk concentrations can be observed, as summarized in Supplementary Figure S4. All Fe-containing CN samples show high densities of accumulated electrons at about 2.95–3.00 eV. Previously reported work (Hu et al., 2014) has suggested that photogenerated electrons are trapped by the Fe-doping sites, promoting efficient separation of photogenerated electrons and holes, resulting in high photocatalytic activity. Accordingly, it may be concluded that the increase in electron-trap density at 2.95–3.00 eV (with regard to that of the pristine CN) is related to the high photoactivity of Fe-doped CN systems (Fe_CN, FeCu_CN, and FeZn_CN) toward acetic acid oxidation. As has been previously reported (Nitta et al., 2018) for patterns of energy-resolved distribution of electron traps (ERDT), the energies might be overestimated as the DOS and density of states at the top of the valence band are negligible. As actual photoexcitation occurs from the high DOS position (energy) part of VB, but not VBT, for titanium(IV) oxide, this energy discrepancy is estimated to be about 0.1–0.2 eV. The higher-energy ERDT might be shallow electron traps located just below CBB. The shallow trap state can capture photoexcited electrons, migrate to the surface, and take part in photocatalysis. Hence, trapped shallow electrons considerably improve photocatalytic activity. In the case of the deep trapped state, these electrons are easily recombined, which leads to poor photocatalytic activity (Choudhury et al., 2014; Ruan et al., 2020; Wang et al., 2021). However, the photocatalytic behavior of Cu_CN cannot be explained by this hypothesis. It might be possible that the higher light absorption intensity in the visible light region extended up to 800 nm (Supplementary Figure S2) makes Cu_CN to be highly light responsive, resulting in the enhanced photocatalytic activity in acetic acid oxidation. As discussed earlier (shown in Figure 4B), the relative intensity of the ESR signal at *g* = 2.0036 corresponds to the number of free unpaired electrons or defects at the localized π-conjugated carbon structure, and that there is a correlation between the number of delocalized unpaired electrons in CN-based materials and the rates of CO_2_ evolution. Such correlations are applicable for classifying these materials as high-performance photocatalysts in terms of CO_2_ evolution rate, i.e., Fe-doped CN (Fe_CN, FeCu_CN, and FeZn_CN) and Cu_CN materials, corresponding to the relatively ESR intensity (*g* = 2.003) of 1.3 and above. The presence of delocalized π-electrons in CN materials described herein, and their relationship with photocatalytic activity for chemical conversion, is in excellent agreement with previous work (Guan and Zhang, 2019; Lin et al., 2021).

## Conclusion

The effects of the introduction of metal dopants on the bulk and surface structure of carbon nitride were studied in relation to their photocatalytic activity in acetic acid oxidation. Powder XRD and ESR techniques confirmed the graphitic stacking and crystallinity in CN-based materials and the presence of free unpaired electrons on the aromatic CN sheets. The enhanced photooxidation rate of acetic acid for Fe-containing CN systems could be the result of the high ET density at the trap state between 2.95 and 3.00 eV and the high number of unpaired electrons available in the CN structure. Relationships between the bulk and surface structure of metal-doped CN materials, as studied by complementary techniques, could be used to classify CN materials as being of high or low photoactivity. Further investigations to probe the interactions between metals and carbon nitride, such as synchrotron measurements at various metal loadings, may allow for high-resolution spectroscopic data to be obtained, which will allow the elucidation of the oxidation state and chemical environment of the metal dopants. Determination of overall degradation byproducts from photocatalytic oxidation of acetic acid by using the metal-doped carbon nitride materials should be carried out to confirm the selectivity of such oxidation.

## Data Availability

The original contributions presented in the study are included in the article/Supplementary Material, further inquiries can be directed to the corresponding authors.
