# Supplementary material for: The Influence of Metal-Doped Graphitic Carbon Nitride on Photocatalytic Conversion of Acetic Acid to Carbon Dioxide
Source: Front Chem. 2022 Mar 23;10:825786. doi: 10.3389/fchem.2022.825786 (PMC8983859; doi:10.3389/fchem.2022.825786)
Supplement: Supplementary file 1 [file DataSheet1.docx]

Supplementary Material

The Influence of Metal doped Graphitic Carbon Nitride on photocatalytic conversion of acetic acid to carbon dioxide

Pichnaree Sakuna^1^, Pradudnet Ketwong^2^, Bunsho Ohtani, ^2^* Jirawat Trakulmututa^1^, Thawanrat Kobkeatthawin^1^, Apanee Luengnaruemitchai,^3^ Siwaporn Meejoo Smith^1^*

^1^ Center of Sustainable Energy and Green Materials and Department of Chemistry, Faculty of Science, Mahidol University, Nakhon Pathom, Thailand

^2^ Institute for Catalysis, Hokkaido University, Hokkaido, Japan

^3^ The Petroleum and Petrochemical College, Chulalongkorn University, Soi Chula 12, Phayathai Road, Bangkok, 10330, Thailand

*** Correspondence:**siwaporn.smi@mahidol.edu and ohtani@cat.hokudai.ac.jp

**Keywords:** carbon nitride, metal doping, photocatalysis, energy-resolved distribution of electron traps (ERDT), electron spin resonance (ESR)

**

**

Supplementary Figure S1. XPS spectra of carbon nitride-based materials showing that the surface concentrations of metals (Fe, Zn, Cu) are lower than the XPS detection limits.








**Supplementary Figure S2.** (Top) Absorption and (Bottom) photoluminescence spectra of as prepared metal doped CN materials and their comparison with pristine CN.





**Supplementary Figure S3.** Nitrogen adsorption-desorption isotherms of pristine CN, single-metal-doped CN and co-metal-doped CN samples.


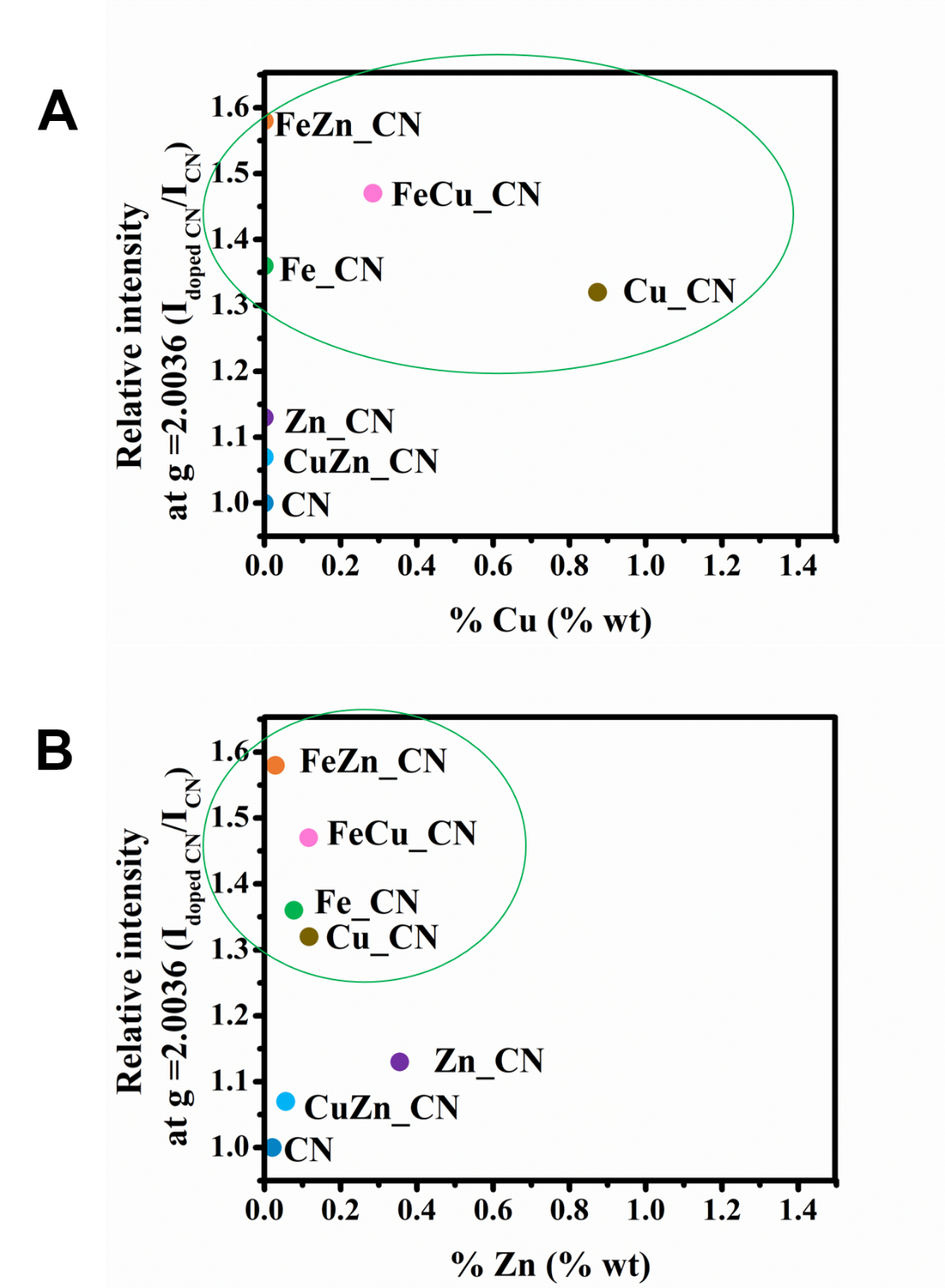


**Supplementary Figure S4.** Correlation between the ESR signal relative intensity at g = 2.0036 and the bulk metal concentration (Cu or Zn) in carbon nitride-based materials. The materials in the green oval gave high CO_2_ evolution rates, as defined in Table 2.
